# Environmental drivers and interaction mechanisms of heavy metal and antibiotic resistome exposed to amoxicillin during aerobic composting

**DOI:** 10.3389/fmicb.2022.1079114

**Published:** 2023-01-04

**Authors:** Ning Liu, Gang Li, Ya Su, Yi Zhao, Jun Ma, Guangqun Huang

**Affiliations:** ^1^Key Laboratory of Urban Environment and Health, Ningbo Urban Environment Observation and Research Station, Institute of Urban Environment, Chinese Academy of Sciences, Xiamen, China; ^2^Zhejiang Key Laboratory of Urban Environmental Processes and Pollution Control, CAS Haixi Industrial Technology Innovation Center in Beilun, Ningbo, China; ^3^Engineering Laboratory for AgroBiomass Recycling and Valorizing, College of Engineering, China Agricultural University, Beijing, China; ^4^School of Water Resources and Environment, China University of Geosciences (Beijing), Beijing, China

**Keywords:** beta-lactam resistance genes, antibiotic resistant bacteria, exchangeable heavy metals, heavy metal resistome, waste utilization

## Abstract

The environmental accumulation and spread of antibiotic resistance pose a major threat to global health. Aerobic composting has become an important hotspot of combined pollution [e.g., antibiotic resistance genes (ARGs) and heavy metals (HMs)] in the process of centralized treatment and resource utilization of manure. However, the interaction mechanisms and environmental drivers of HMs resistome (MRGs), antibiotic resistance (genotype and phenotype), and microbiome during aerobic composting under the widely used amoxicillin (AMX) selection pressure are still poorly understood. Here, we investigated the dynamics of HMs bioavailability and their MRGs, AMX-resistant bacteria (ARB) and antibiotic resistome (ARGs and *intI1*), and bacterial community to decipher the impact mechanism of AMX by conducting aerobic composting experiments. We detected higher exchangeable HMs and MRGs in the AMX group than the control group, especially for the *czrC* gene, indicating that AMX exposure may inhibit HMs passivation and promote some MRGs. The presence of AMX significantly altered bacterial community composition and AMX-resistant and -sensitive bacterial structures, elevating antibiotic resistome and its potential transmission risks, in which the proportions of ARB and *intI1* were greatly increased to 148- and 11.6-fold compared to the control group. Proteobacteria and Actinobacteria were significant biomarkers of AMX exposure and may be critical in promoting bacterial resistance development. *S0134_terrestrial_group* was significantly negatively correlated with *bla_TEM_* and *czrC* genes, which might play a role in the elimination of some ARGs and MRGs. Except for the basic physicochemical (MC, C/N, and pH) and nutritional indicators (NO_3_^−^-N, NH_4_^+^-N), Bio-Cu may be an important environmental driver regulating bacterial resistance during composting. These findings suggested the importance of the interaction mechanism of combined pollution and its synergistic treatment during aerobic composting need to be emphasized.

## Introduction

With the overuse, misuse, and abuse of antibiotics in modern medicine and the livestock industry in the last decades (Yao L. et al., 2020; Zhang et al., 2020; Zou et al., 2020; Wang et al., 2021), antimicrobial resistance has developed into one of the most urgent public health crises in recent years (Zhu et al., 2017a; Vos et al., 2019; Joshi and Kim, 2020; Dai et al., 2022). According to statistics, the global defined daily doses of antibiotics reached 34.8 billion in 2015 and have increased by 65% since 2000 (Zhao R. et al., 2019). Amoxicillin (AMX) is currently the most important and frequently used broad-spectrum β-lactam antibiotic, accounting for 50–70% of total antibiotic consumption globally (Chen et al., 2017; Liu et al., 2018). Specifically, AMX is the most commonly used human and animal antibiotic among 36 different drug types in China (Liu et al., 2019), with doses up to 500 mg/kg used in pig feed (Liu et al., 2018). However, antibiotics usage in animal husbandry (Zhang et al., 2017a) can shift animal microbiome, enrich antibiotic resistance genes (ARGs), and subsequently transfer to livestock manure and its products (Qiu et al., 2022). Hence, livestock production is considered an important hotspot and key point-source of antibiotic resistant bacteria (ARB) and ARGs dissemination (Youngquist et al., 2016; Awasthi et al., 2019; Deng et al., 2020; Lu et al., 2020; Tang et al., 2020). Previous studies have shown that β-lactam resistance genes are the top three types of ARGs in manure samples from 17 dairy farms in Shanxi Province, China (Zhou et al., 2016; Liu et al., 2018). The Class I integrase gene (*intI1*), which gene cassette usually contains ARGs (Zhang et al., 2017a; Zhang R. et al., 2019), can mediate horizontal gene transfer (HGT) thereby facilitating ARGs spread among microorganisms (Yin et al., 2017; Zhang J. et al., 2019; Wang et al., 2023). Due to persistent environmental selection of antibiotic residue and fecal contamination containing antimicrobial resistant determinants, it is anticipated that about 300 million people will die prematurely by 2055 worldwide (Joshi and Kim, 2020).

It is noteworthy that heavy metals (HMs), such as copper (Cu) and zinc (Zn), were often used as important feed additives to promote the growth of livestock (Zhang J. et al., 2019; Zhang et al., 2020). However, due to the poor absorption and difficult degradation of HMs (Yin et al., 2017; Zhang et al., 2017b), they usually coexist with antibiotics and other harmful substances (Imran et al., 2019; Wang R.Z. et al., 2020; Yao N. et al., 2020) in breeding wastes (Awasthi et al., 2019; Zhang R. et al., 2019; Chen et al., 2020) and compost products (Guo et al., 2019; Deng et al., 2020; Zhang et al., 2020). These environmental pollutants (HMs, antibiotics, etc.) can release (co-)selection pressure, induce HMs resistome (MRGs) development and mobile genetic elements (including *intI1*) mediated HGT (Imran et al., 2019; Zhao Y. et al., 2019; Lu et al., 2020) and accelerate the spread of bacterial resistance (Zhao et al., 2017; Vos et al., 2019), thereby seriously threatening the environmental health (Guo et al., 2019; Imran et al., 2019; Yang et al., 2019). Worse still, even HMs of low concentration has high persistence (Yin et al., 2017; Vos et al., 2019) and strong selective pressure of antibiotic resistance in certain cases (Imran et al., 2019; Zhang J. et al., 2019), among which Cu was shown to be the strongest ability to promote ARGs conjugate transfer (Ji et al., 2012; Guo et al., 2019; Zhang R. et al., 2019).

Pig manure is a typical microenvironment contaminated with antibiotics and HMs (Zhang et al., 2017b; Zhang J. et al., 2019; Zhang R. et al., 2019), where the concentration of HMs is several times that of antibiotics (Awasthi et al., 2019; Imran et al., 2019). It has been reported that aerobic composting has the potential to reduce the bioavailability of HMs (Yin et al., 2017; Deng et al., 2020), antibiotic concentrations, and their drug resistance (Youngquist et al., 2016; Zhou et al., 2016; Awasthi et al., 2019), while utilizing livestock manure as resources (Liu et al., 2017a,b, 2018; Guo et al., 2019; Kumar Awasthi et al., 2019; Qiu et al., 2019; Tang et al., 2020). However, the removal effect of composting will largely vary depending on the composting environment, process conditions, and various pollutants (Zhang J. et al., 2019; Zhang et al., 2020). Up to date, many studies on the removal (Youngquist et al., 2016; Liu et al., 2018; Awasthi et al., 2019; Tang et al., 2020; Zhang et al., 2020) and effects (Meng et al., 2015; Qian et al., 2016; Yin et al., 2017; Liu et al., 2019; Deng et al., 2020) of various antibiotics or HMs during aerobic composting have been made. However, there is still a lack of research on the dynamics and interaction mechanisms of HMs resistome, AMX bacterial resistance (genotype and phenotype), and microbiome during aerobic composting under the widely used AMX selection pressure. Therefore, more studies are needed to decipher the impact mechanism of AMX on various complex pollutants in the process of fecal aerobic composting and the risk of bacterial resistance.

Thus, the objectives of our study were (1) to characterize the dynamics of HMs bioavailability and their resistome (MRGs) with or without AMX exposure, (2) to explore the effects of AMX selection pressure on AMX bacterial resistance (ARB) and antibiotic resistome (ARGs and *intI1*), and (3) to decipher the environmental drivers and interaction mechanisms of bacterial taxonomic and functional (ARGs, MRGs, *intI1*, etc.) community composition during composting. Our findings will provide insights into organic waste resource utilization and pollutant risk control mechanisms underpinning the cleaner production of intensive animal husbandry and better development of organic circular agriculture.

## Materials and methods

### Materials and chemicals

Pig manure was obtained from a large-scale pig farm in Shunyi District (Beijing, China), with prior assurance that no antibiotics were applied to the sampled pigs during the breeding process. Wheat straw was collected from Shangzhuang Experimental Station of China Agricultural University and cut into 3–5 cm segments (Liu et al., 2017a,b, 2019). AMX (98%) was gained from Huamaike Biological Technology Company, Ltd. (Beijing, China). The chemicals used in HM extraction were of analytical grade.

### Experimental design

In this study, two groups of fresh pig manure (about 5.2 kg) were fully mixed with 100 mg/kg (dry weight) AMX aqueous solution or an equivalent amount of ultrapure water (control), and then a certain amount of wheat straw and ultrapure water were mixed uniformly, according to the ratio of total carbon and total nitrogen (C/N) of 20:1 and moisture content (MC) of 65% (Liu et al., 2019). After being completely mixed and equilibrated for about 6 h (Qian et al., 2016; Liu et al., 2019), the two groups of initial compost mixture were placed in two parallel sets of in-laboratory aerobic composting reactor systems (Supplementary Figure S1), as the experimental group (AMX group) and the control group without AMX (CK group). Both groups of reactors were continuously ventilated by 0.2 L/(kg-*VS*-min). The basic physicochemical properties of initial composting materials have been determined and listed in Supplementary Table S1. The above settings of composting conditions and AMX concentration were based on the existing reports and our previous studies (Kotzerke et al., 2011; Liu et al., 2018, 2019).

According to the aerobic composting periods (Liu et al., 2018), about 120 g of the compost mixture was sampled on days 0, 2, 4, 6, 9, 15, and 21, and the basic physicochemical, biological, and heavy metal-related indicators were determined. Furthermore, the viable counts of AMX-resistant bacteria and total culturable bacteria (TCB) were performed immediately on the day of sampling. The presence of AMX resistance genes (*bla_TEM_*, *bla_VIM_*), Cu resistance gene (*copA*), Zn resistance gene (*czrC*), and *intI1* were determined by real-time fluorescence quantitative PCR (qPCR) for day 0, 2, 6, 15, and 21 samples. Combined with the existing reports and our previous studies, *TEM-* and *VIM*-type β-lactam resistance genes, as the most common and highly abundant ARGs in animal breeding and clinical environments, can significantly enhance the zoonotic potential (Pomba et al., 2004). Together with *copA-* and *czrC*-type MRGs, they are often used as indicator genes of bacterial resistance contamination (Narciso-da-Rocha et al., 2014). Moreover, *intI1* is widely used as an important universal marker for human pollutants (Gillings et al., 2015; Yin et al., 2017). The bacterial community of compost on days 2, 6, and 15 were analyzed according to different representative composting stages based on 16S rRNA gene sequencing. Samples used for molecular biological determinations were first lyophilized with a vacuum freeze dryer (Martin Christ Alpha 1–2 LD plus, Germany) for the same low MC, then ground with a 1-mm sieve and frozen at −80°C for DNA extraction. Other samples were kept at −20°C for physicochemical parameters analysis. The compost piles were fully mixed manually before and after each sampling.

### Measurement and analysis methods

#### Basic physicochemical and biological indicators

Temperature sensor systems were used to monitor the temperature of the piles and ambient temperature in real-time during composting. Organic matter (OM), MC, C/N, pH, NH_4_^+^-N, and NO_3_^−^-N were determined concerning the existing methods (Liu et al., 2017a,b, 2019). Since the level of mobility is an important indicator to measure the potential risk of HMs in the environment (Nemati et al., 2011), exchangeable HMs have also attracted attention as the most active and bioavailable high-risk metal forms. Therefore, the most bioavailable and risky exchangeable copper (bio-Cu) and exchangeable zinc (bio-Zn) were detected according to the improved BCR sequential extraction method (Nemati et al., 2011).

#### Viable counts of ARB and TCB

According to the maximum value of the bacterial Minimum Inhibition Concentration, AMX-resistant bacteria and TCB were counted on R2A medium with and without 32 mg/L AMX by referring to previous experience (Liu et al., 2020). The colony formation unit (CFU) per gram was calculated by the formula (1). All measurements in this study were performed in duplicate.

(1) $$\mathrm{CFU}\left(\frac{1}{\mathrm{ml}}\right)=\frac{\mathrm{colony count}}{\mathrm{sample volume}\left(\mathrm{ml}\right)\times \mathrm{dilutionrate}}$$

#### DNA extraction and qPCR

According to the manufacturer’s instructions, a Soil Genomic DNA Extraction Kit (Kangwei Century, China) was used to extract DNA from composting samples. After the design of related primers (Supplementary Table S2), the presence of two β-lactam resistance genes (*bla_TEM_*, *bla_VIM_*), two MRGs (*copA*, *czrC*), and *intI1* were determined by standard PCR (Liu et al., 2019). The PCR products were examined by 1% (w/v) agarose gel electrophoresis after the reaction, and the detected genes were quantified using the ABI 7500 Real-Time PCR System (Applied Biosystems, United States). Absolute abundances of genes were expressed as copy number per gram of dry compost.

#### Bacterial 16S rRNA gene high-throughput sequencing

16S rRNA gene sequencing was conducted with the Illumina MiSeq platform, and the 16S V3-V4 region was amplified using primers U341F (ACTCCTACGGGAGGCAGCAG) and U806R (GGACTACHVGGGTWTCTAAT). USEARCH was used for quality control of raw data, and UPARSE was used for clustering of qualified sequences with 97% similarity to form operational classification unit (OTU).

### Statistical analysis

Repeated measurements were expressed as mean ± standard deviation (SD, indicated by the error bar in the figures). Excel 2016 (Microsoft, United States), SPSS 25 (IBM, United States), and OriginProV.8.5 SR1 (OriginLab Corp., United States) were used for basic statistical analysis. Shannon-Wiener curves, principal coordinates analysis (PCoA), heatmap, correlation analysis, and genus-level phylogenetic tree were performed by R3.4.1 software. Redundancy analysis (RDA) was carried out based on CANOCO 5.0. Correlation network analysis (Spearman, *r* > 0.6, *p* < 0.05) was conducted by R3.4.1 software and Gephi0.9.3 software.

## Results and discussion

### Changes of exchangeable HMs and their resistome (MRGs) during composting

**Figure 1:**
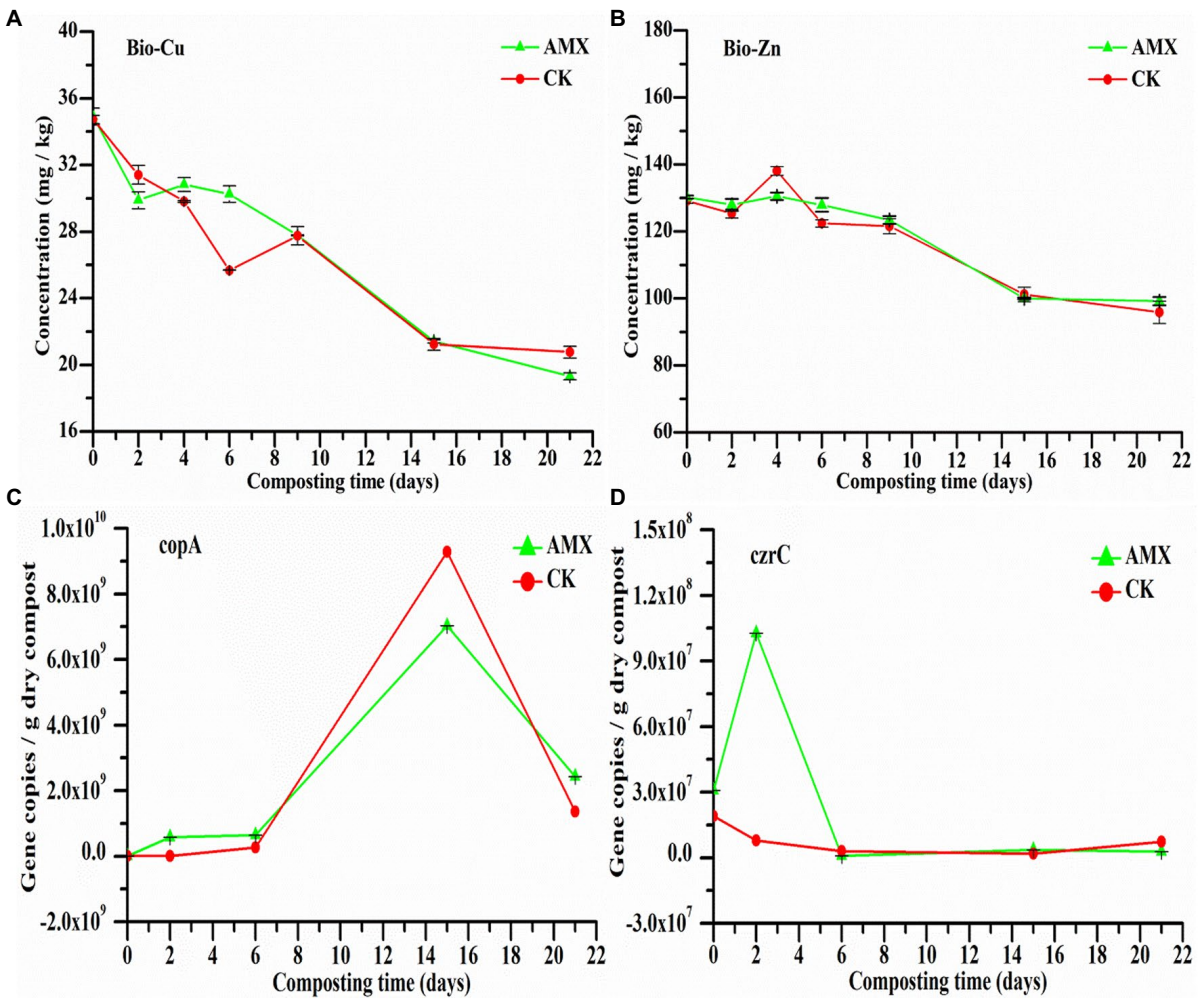
Dynamic changes of **(A)** exchangeable Cu (bio-Cu), **(B)** exchangeable Zn (bio-Zn), **(C)** Cu resistance gene (*copA*), and **(D)** Zn resistance gene (*czrC*) during composting.

As important indicators of environmental HMs potential risks, exchangeable HMs (bio-Cu and bio-Zn) can sensitively reflect the dynamic changes of HM morphology and toxicity during composting. The concentrations of bio-Cu and bio-Zn in the two groups leveled off after first decreasing during composting, presenting a relatively consistent change trend (Figures 1A,B). The results reflected that aerobic composting treatment will reduce the migration rate and bioavailability of Cu and Zn to a certain extent, thereby effectively reducing the toxicity and potential risks of HMs in the products. Comparatively, the concentrations of bio-Cu and bio-Zn in the AMX group were slightly higher than those in the CK group during the early stage of composting. This difference indicated that the addition of AMX likely hindered the passivation process of HMs and, thus, increased the risks of HMs contamination and the co-selection pressure of inducing bacterial resistance, which was also confirmed by previous reports (Song et al., 2017; Vos et al., 2019).

We found that the abundance of *copA* and *czrC* in the AMX group was higher at the initial stage of composting (Figures 1C,D), especially the difference between the two groups of *czrC* was the most significant (*p* < 0.05, *t*-test), further illustrating the vital role of AMX selective pressure on the promotion of HM resistance and adaptation cost of MRGs-carrying bacteria (Imran et al., 2019; Zhang et al., 2020). Notably, AMX may have a stronger promotion effect on some Zn resistome. In the late stage of composting, the abundance of *copA* in the two groups increased rapidly then fell, while *czrC* of the two groups was very low and stable, probably because *copA*-carrying bacteria mainly dominated the composting maturity stage and were strongly affected by microbial succession at this stage. According to Figure 1, AMX selective pressure may significantly inhibit the passivation of HMs while promoting the development of some MRGs, thereby raising the potential risk of HM toxicity and bacterial resistance in the environment.

### Changes of AMX bacterial resistance (phenotype and genotype) during composting

#### AMX-resistant bacteria and their proportion of resistance

**Figure 2:**
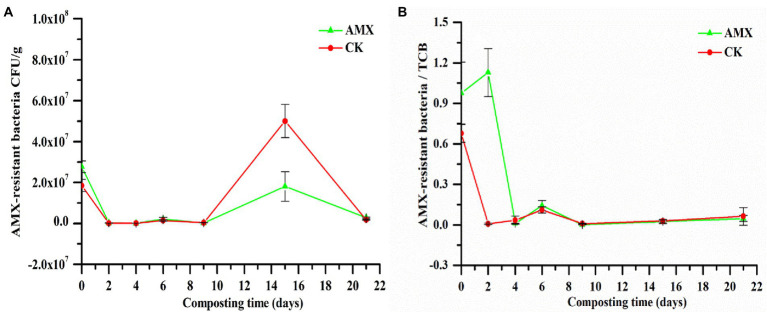
Dynamic changes of **(A)** AMX-resistant bacteria and **(B)** AMX-resistant bacteria / total culturable bacteria (TCB) during composting.

As shown in Figure 2A, the number of AMX-resistant bacteria in the two groups fell sharply with the rapid increase in compost temperature (Supplementary Figure S2A) on days 0–2, which also restricted TCB (Supplementary Figure S2B). The temperature of compost piles on days 3–9 decreased slowly, with a significant recovery in the middle period, which resulted in a slight increase in the number of AMX-resistant bacteria and TCB in the two groups. As the compost temperature gradually decreased toward ambient temperature (days 10–21), the composting microenvironment became more suitable for the growth and reproduction of microorganisms. Therefore, a sharp increase in the number of AMX-resistant bacteria and TCB was observed, followed by a decrease due to nutrient deficiency.

The proportion of AMX-resistant bacteria in the AMX group in the early stage of composting were significantly higher than those in the CK group (days 0–4; Figure 2B), implying the influence of AMX selective pressure on the enhancement of AMX bacterial resistance (Vos et al., 2019; Zou et al., 2020). However, in the later stage of composting, the number of AMX-resistant bacteria and TCB in the AMX group was significantly lower (*p* < 0.01, *t*-test) than that in the CK group (Figure 2A; Supplementary Figure S2B), while the proportion of AMX resistance (Figure 2B) was similar between the two groups, suggesting the inhibitory effect of AMX addition on microbial growth and reproduction (Liu et al., 2019).

#### ARGs and intI1 of the compost piles

**Figure 3:**
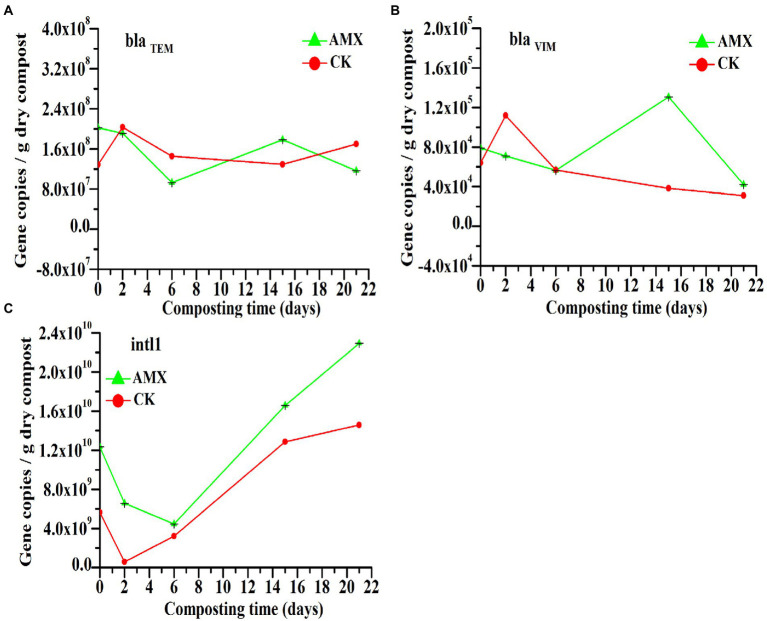
Dynamic changes in the copy number of **(A)** *bla_TEM_*, **(B)** *bla_VIM_*, and **(C)** *intI1* during composting.

According to Figures 3A,B, the *bla_TEM_* and *bla_VIM_* gene abundances in the AMX treatment were initially higher than those of the CK treatment. Both genes in the AMX group experienced a tendency to decline first, followed by an increase, and then a gradual decrease, which may be due to the dual effect of the high-temperature environment (Supplementary Figure S2A) and antibiotic selective pressure in the early stage of composting. With the piles’ temperature decrease in the late stage of composting and the long-term co-selection of AMX-HM pollution (Imran et al., 2019; Wang R.Z. et al., 2020; Yao N. et al., 2020), the abundance of the two β-lactam resistance genes in the AMX group obviously increased compared with the CK group.

As shown in Figure 3C, the absolute abundance of *intI1* gene in the two groups changed similarly during composting, both demonstrating an initially decreasing trend followed by an increase. Comparatively, the abundance of *intI1* in the AMX group was always 1.3–11.6 times greater than that in the CK group, even at the day 0, and maintained a rapid growth trend in the late stage of composting. Compared with *bla_TEM_* and *bla_VIM_*, *intI1* was significantly increased during the equilibration phase before the start of composting (day 0), indicating that it would be greatly increased in a very short period of time and persistently affected by AMX exposure, which may be a non-negligible problem in the spread of bacterial resistance. Furthermore, while the high temperature of the thermophilic phase greatly reduced the number of bacteria (Supplementary Figure S2) and *intI1* gene in both groups, the co-selection pressure of AMX and HMs still provided a strong selective advantage for their resistant bacteria (Figure 2) and resistome (Figures 1C,D, 3) in the AMX group. This further confirmed the crucial role of antibiotics in promoting the development of bacterial resistance (antibiotics, HMs, etc.) and its transmission risk (Imran et al., 2019).

### Changes of bacterial communities during composting

**Figure 4:**
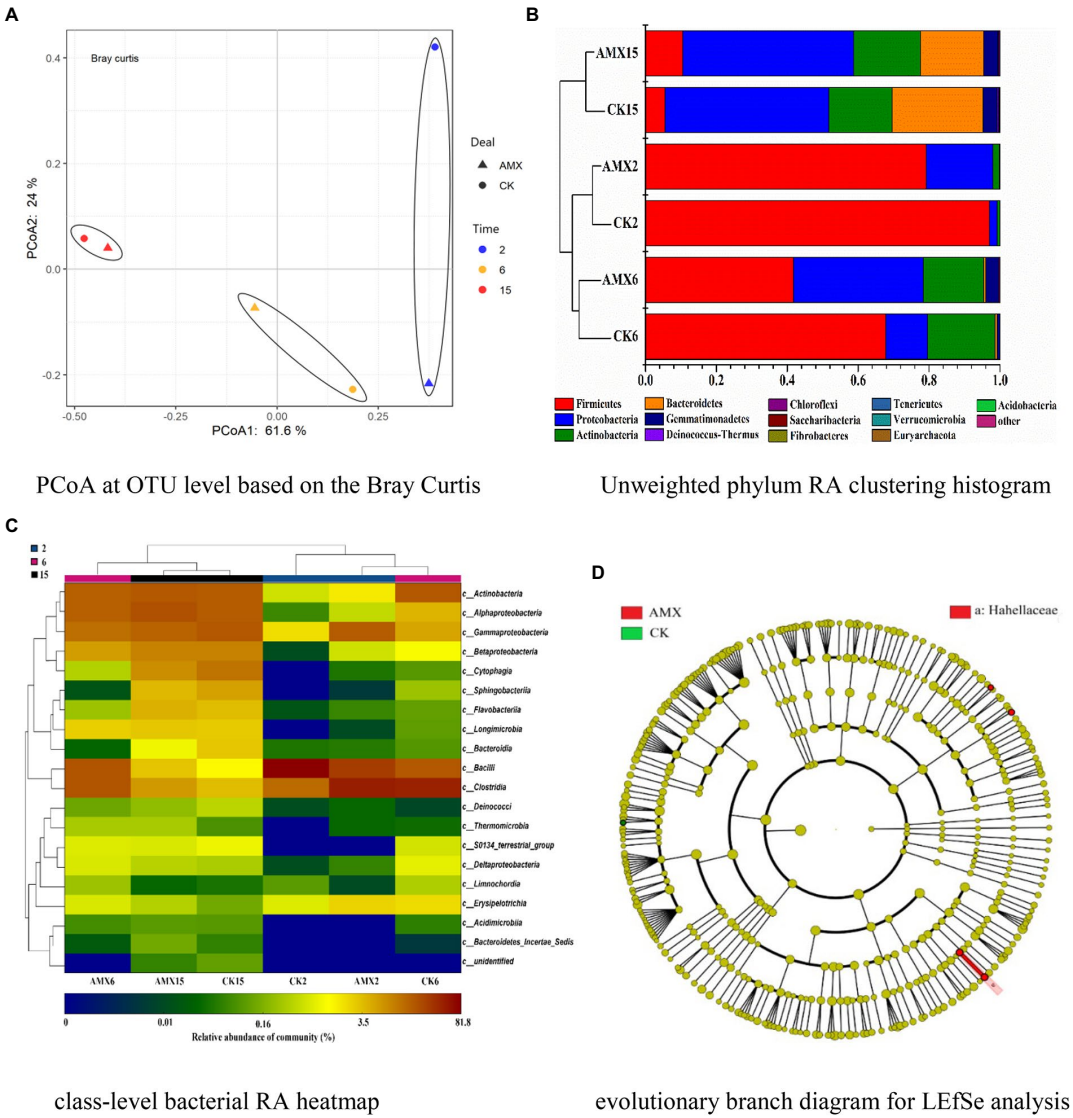
Features of bacterial community during composting. **(A)** PCoA at OTU level based on the Bray Curtis. **(B)** Unweighted phylum RA clustering histogram. **(C)** Class-level bacterial RA heatmap. **(D)** Evolutionary branch diagram for LEfSe analysis.

A total of 397 operational taxonomic units (OTUs) were detected in compost samples. According to the Shannon-Wiener curves of the compost samples (Supplementary Figure S3), the sequencing data were sufficient to reflect the vast majority of microbial information in all samples. After leveling the minimum sequence number of the samples, alpha diversity analysis was performed on the two groups of compost samples (Supplementary Figure S4). The three diversity indices of the AMX group were higher than those of the CK group as a whole, confirming that the bacterial community of the AMX group was relatively rich and uniform with higher community diversity (Supplementary Figures S4A–C). Moreover, there was a greater difference in species lineage among samples in the CK group when considering species abundance and evolutionary distance (Supplementary Figure S4D). In summary, although AMX selective pressure reduced the absolute abundances of culturable bacteria (Supplementary Figure S2B), the richness and evenness of the bacterial community were relatively high (Supplementary Figure S4), which may indicate the effect of AMX on the alteration of microbial community structure during composting and the selective pressure on AMX-sensitive and -resistant bacteria. According to the PCoA, PC1 and PC2 accounted for 85.6% of the total variation of the bacterial community (Figure 4A). Principal coordinates analysis of the bacterial community revealed that the samples of different composting periods were clearly separated along the PCo1 axis, and some clustering of composting samples from the same period was also observed. The difference in bacterial community composition between the two groups decreased gradually as the number of composting days increased, illustrating that the impact of the composting periods and habitat on the bacterial community structure was particularly significant (Yin et al., 2017). Furthermore, AMX addition significantly affected the bacterial community structure in the early stage of composting, which is consistent with the above results (Supplementary Figure S4).

It can be seen from Figures 4B,C that the bacterial community of the two groups continued to change during composting, while the differences in community composition gradually decreased. Among the communities, Firmicutes, Proteobacteria, Actinobacteria, and Bacteroidetes were the dominant phylum bacteria during the whole composting process (Figure 4B), accounting for 95.2–99.9% of the bacterial 16S rRNA gene sequences (Supplementary Figure S5). As shown in Figure 4B, the relative abundance (RA) of Firmicutes was the highest in the early stage of composting and the RA in the AMX group was lower than that in the CK group, but decreased rapidly as the compost matured. This may be since Firmicutes, beneficial bacteria for promoting cellulose degradation, widely exist in animal intestines, compost, and soil environment, and dominate the heating and thermophilic phases of composting (Yin et al., 2017; Liu et al., 2019). Moreover, high concentrations of antibiotics will significantly inhibit their growth and reproduction (Qian et al., 2016; Guo et al., 2017; Liu et al., 2019), resulting in a significant reduction of Firmicutes RA in the AMX group. According to Figure 4B, RA of Bacteroides increased significantly in the mature composting stage of the two groups and was relatively higher in the CK group. In Figure 4C, it can be seen that Sphingobacteriia, Flavobacteriia, and Bacteroidia (which belong to Bacteroides) also displayed the same regularities and cluster. Bacteroides are a kind of bacteria attributed to the degradation of high-molecular-weight compounds and the growth of supporting materials, which mainly grow in the middle and late stages of composting (Zhu et al., 2017b; Liu et al., 2019). The RA of Proteobacteria and Actinobacteria (most notably in Proteobacteria) in the AMX group increased with composting maturity and was significantly higher than that in the CK group during the early stage of composting (Figure 4B). This large difference may be attributed to the dominance of both bacteria in the cooling and mature stages of composting, where the abundance of Actinobacteria is mostly used to mark the degree of maturity (Liu et al., 2019; Zhang et al., 2020). Previous studies have shown that both bacteria have a significant positive correlation with ARGs (Yin et al., 2017), and their RA will also be significantly increased in the presence of high concentrations of antibiotics (Guo et al., 2017). Besides, the driving force of the increased resistance in the compost was mainly attributed to Proteobacteria (Meng et al., 2015), in which γ-Proteobacteria has a strong resistance potential to high-concentration antibiotics (Zhu et al., 2017b). Moreover, β-Proteobacteria is considered to be the original source of *intI1*, which has become the core of antibiotic resistance due to its ability to capture and express multiple ARGs (Guo et al., 2017; Zhu et al., 2017b). The above conclusions were also further confirmed in Figure 4C, where the variation rules of α, β, γ-Proteobacteria, and Actinobacteria were similar and clustered with each other. Therefore, the high concentration of AMX added to the experimental group can selectively inhibit the growth and reproduction of sensitive bacteria, such as Firmicutes. This provided a more favorable living environment for drug-resistant Proteobacteria and Actinobacteria, thus affecting and changing the bacterial community structure and drug resistance level during composting.

LEfSe analysis (threshold set to 2) was used to further reveal the differences between the two groups of bacterial community during composting (Figure 4D). Compared with the CK group, the species with significantly different abundance in the AMX group were Hahellaceae, Hahella, Enteractinococcus, and Marmoricola (Supplementary Figure S6), where the former two species belong to γ-Proteobacteria and the latter two to Actinobacteria. Proteobacteria is the main host of ARGs and has a high tolerance to antibiotics, while Actinobacteria is the main producer of antibiotics and usually carries a variety of ARGs (Qian et al., 2016; Zhang et al., 2020). Therefore, this finding was consistent with the relevant conclusions in Figures 4B,C and previous literature reports, further confirming the significant role of AMX in reshaping the structure of environmental microbial communities and promoting bacterial resistance. Studies have shown that Enteractinococcus is a type of Micrococcaceae commonly found in animal feces (Cao et al., 2012), while Marmoricola is the gram-positive aerobic bacteria belonging to the Nocardiaceae (Habib et al., 2020). Both of them have been reported to exist in the aerobic composting environment of livestock feces as the main Actinobacteria (Chen et al., 2015).

### Correlation analysis of the compost piles

**Figure 5:**
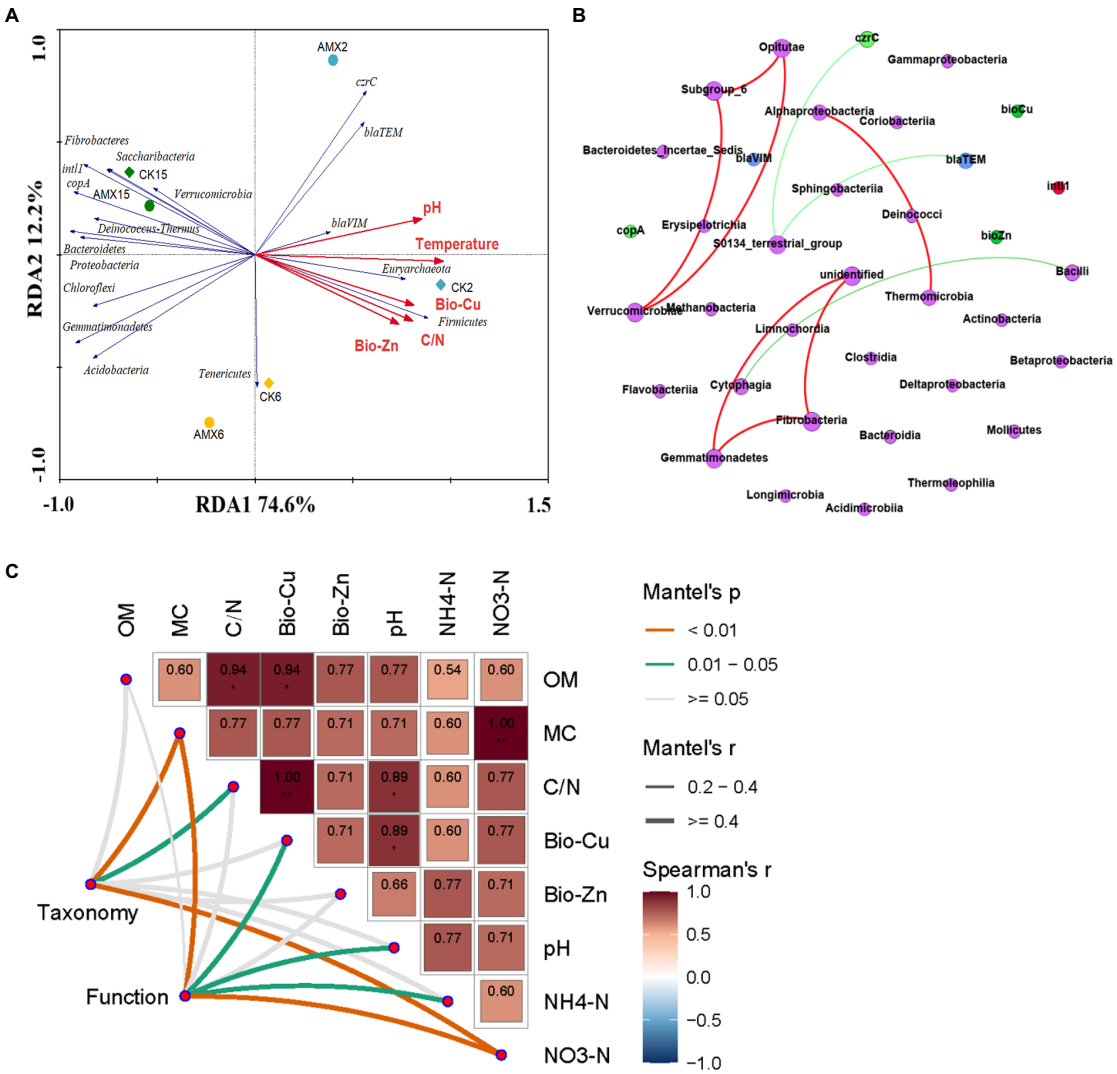
Environmental drivers of compost microbial community composition: **(A)** RDA of the relationships between the main bacterial phylum (more than 1% of the total bacterial abundance, blue arrows) and environmental factors (red arrows), **(B)** network analysis of the relationships among class-level bacteria (purple nodes), ARGs (blue nodes), MRGs (light green nodes), HMs (green nodes), and *intI1* (red nodes), and **(C)** pairwise comparisons of environmental factors were shown, with color gradients representing Spearman’s correlation coefficients. Taxonomic (based on family-level bacteria) and functional (based on ARGs, MRGs, *intI1*, ARB, TCB, and ARB/TCB) community composition was related to each environmental factor by Mantel tests. Edge width denotes the Mantel’s *r* statistic for the corresponding distance correlations, and edge color represents the statistical significance.

Redundancy analysis was carried out to further reveal the correlation between the bacterial community structure and its function and environmental factors during composting, which can explain 86.8% of the total species variation (Figure 5A). Differences in the bacterial community structure gradually reduced with the extension of composting time, which intuitively reflected the severe succession and stable formation of the bacterial community structure during composting. A noteworthy correlation was observed between the thermophilic period of composting and the dynamic changes in the basic physiochemical indicators, exchangeable HMs, β-lactam resistance genes, *czrC* gene, and Firmicutes. Proteobacteria, Actinobacteria, and Bacteroidetes were mainly distributed in the composting maturation stage and were directly related to the dynamic changes of *copA* gene and *intI1* gene. This correlation demonstrated the significant impact of the composting stages (especially the thermophilic period) and bacterial community structure on the composting microenvironment, HM forms, and resistance genes. Furthermore, it can also be suggested that Proteobacteria, Actinobacteria, and Bacteroidetes were likely the main factors directly affecting the development and spread of HM resistance and antibiotic resistance during composting.

To further verify the interaction pattern between the microbial communities in the two groups of different composting environments, the relationships among class-level bacteria, ARGs, MRGs, Bio-HMs, and *intI1* were revealed by network analysis (Figure 5B). More positive edges (red, 7) than negative edges (green, 3) were detected in a network of the compost. Seven different class-level bacteria (except for unidentified) had a significant positive correlation with each other (*p* < 0.05), which was consistent with the results of the RDA analysis. *Bacilli* (belonging to Firmicutes) and *Cytophagia* (belonging to Bacteroidetes) were significantly negatively correlated (*p* < 0.05), which were the dominant bacterial communities in the early composting period and the late composting periods, respectively, (Figure 4B). *S0134_terrestrial_group*, which belongs to the Gemmatimonadetes, was significantly negatively correlated with *bla_TEM_* gene and *czrC* gene (*p* < 0.05). Combined with Figure 4B, Gemmatimonadetes were mainly detected in the middle and late stages of compost samples (AMX6, AMX15, and CK15), and probably play an important role in the reduction of *bla_TEM_* gene and *czrC* gene. Therefore, it is recommended in future studies to further explore the role of Gemmatimonadetes in removing other ARGs and MRGs during aerobic composting, especially β-lactams and zinc.

Moreover, to identify environmental drivers in our study (Sunagawa et al., 2015), we correlated distance-corrected dissimilarities of taxonomic and functional community composition with those of compost environmental factors (Figure 5C). Overall, MC and NO_3_^−^-N were the strongest correlates of both taxonomic and functional composition during composting, while no significant correlations were found for OM and Bio-Zn. Bio-Cu, pH, and NH_4_^+^-N were only weakly correlated with functional community, as well as C/N with taxonomic composition, and the other correlations were not statistically significant (Figure 5C). It can be seen that, except for the basic physicochemical (MC, C/N, and pH) and nutritional indicators (NO_3_^−^-N, NH_4_^+^-N), Bio-Cu may be an important environmental driver affecting taxonomic and functional community composition in composting environments, as well as an important potential factor regulating bacterial resistance.

## Conclusion

Our study found that aerobic composting could effectively decrease HMs bioavailability, which may alleviate the combined pollutions and (co-)selection pressure of AMX and HMs. AMX selective pressure played an important role in inhibiting HMs passivation and bacterial growth, shifting bacterial community composition and AMX-sensitive and -resistant bacteria structures, and increasing some MRGs, AMX bacterial resistance, and their potential risks, especially for *czrC* and *intI1*. Proteobacteria and Actinobacteria, as significant biomarkers of AMX exposure, may be critical in promoting the development and spread of bacterial resistance. *S0134_terrestrial_group* was significantly negatively correlated with *bla_TEM_* and *czrC* gene, which may be critical in reducing some ARGs and MRGs. Except for the basic physicochemical (MC, C/N, and pH) and nutritional indicators (NO_3_^−^-N, NH_4_^+^-N) during composting, Bio-Cu may be an important environmental driver affecting taxonomic and functional community composition and regulating bacterial resistance. Therefore, it is necessary to further explore the efficient removal technology of combined pollutants and the interaction mechanisms between important microorganisms and contaminants in the process of composting, to promote the harmless treatment, resource recovery, and agricultural utilization of organic waste.

## Data availability statement

The data presented in the study are deposited in the Genome Sequence Archive (Genomics, Proteomics & Bioinformatics 2021) repository, accession number CRA009005 that are publicly accessible at: https://ngdc.cncb.ac.cn/gsa.

## Author contributions

NL: conceptualization, methodology, investigation, software, formal analysis, writing—original draft, visualization, and writing—review and editing. GL: investigation, methodology, and review and editing. YS: investigation and visualization. YZ: article revision and grammar correction. JM: methodology, review and editing, and supervision. GH: resources, writing—review and editing, supervision, and funding acquisition. All authors contributed to the article and approved the submitted version.

## Funding

This work was financially supported by the National Natural Science Foundation of China (31771684, 42090063, and 42107407), Ningbo Science and Technology Plan Project (2021S030) and the Project funded by China Postdoctoral Science Foundation (2022M713076).

## Conflict of interest

The authors declare that the research was conducted in the absence of any commercial or financial relationships that could be construed as a potential conflict of interest.

## Publisher’s note

All claims expressed in this article are solely those of the authors and do not necessarily represent those of their affiliated organizations, or those of the publisher, the editors and the reviewers. Any product that may be evaluated in this article, or claim that may be made by its manufacturer, is not guaranteed or endorsed by the publisher.
