# Supplementary material for: Environmental drivers and interaction mechanisms of heavy metal and antibiotic resistome exposed to amoxicillin during aerobic composting
Source: Front Microbiol. 2023 Jan 4;13:1079114. doi: 10.3389/fmicb.2022.1079114 (PMC9845726; doi:10.3389/fmicb.2022.1079114)
Supplement: Supplementary file 1 [file Data_Sheet_1.docx]

**Supplementary Material**

**Environmental drivers and interaction mechanisms of heavy metal and antibiotic resistome exposed to amoxicillin during aerobic composting**

Ning Liu ^a, b^, Gang Li ^a, b^, Ya Su ^c^, Yi Zhao ^d^, Jun Ma^a, b*^, Guangqun Huang^c*^

^a^ Key Laboratory of Urban Environment and Health, Ningbo Urban Environment Observation and Research Station, Institute of Urban Environment, Chinese Academy of Sciences, Xiamen 361021, China.

^b^ Zhejiang Key Laboratory of Urban Environmental Processes and Pollution Control, CAS Haixi Industrial Technology Innovation Center in Beilun, Ningbo 315830, China.

^c^ Engineering Laboratory for AgroBiomass Recycling & Valorizing, College of Engineering, China Agricultural University, Beijing 100083, China.

^d^ School of Water Resources and Environment, China University of Geosciences (Beijing), Beijing 100083, China.

Corresponding author：

Jun Ma, E-mail: [jma@iue.ac.cn;](mailto:jma@iue.ac.cn;)

Guangqun Huang, E-mail: [huanggq@cau.edu.cn](mailto:huanggq@cau.edu.cn)

1. **Tables：**

Table S1 Basic physicochemical properties of the composting materials.

| Composting materials ^a^ | MC (%) ^b^ | OM ^c^ | C/N ^c^ | Cu ^c^ | Zn ^c^ | AMX (μg / kg) ^c^ |
| --- | --- | --- | --- | --- | --- | --- |
| PM | 73.50±0.02 | 79.77±0.01 | 15.14±0.06 | 237.5±53.03 | 373.9±63.64 | BLLOQ ^d^ |
| WS | 6.70±0.01 | 94.11±0.14 | 54.05±0.92 | 7.35±0.78 | 22.25±5.73 | BLLOQ ^d^ |
| AMX group | 64.71±0.62 | 83.83±0.07 | 18.13±0.07 | 282.5±38.89 | 456.4±24.75 | BLLOQ ^d^ |
| CK group | 60.45±0.69 | 85.66±0.09 | 19.68±0.13 | 253±12.73 | 403.4±34.65 | BLLOQ ^d^ |

^a^: Data are expressed as the means ± standard deviations of the duplicate measurements

^b^: Moisture content (MC), measurement based on wet weight.

^c^: Organic matter (OM), ratio of total carbon and total nitrogen (C/N), content of Amoxicillin (AMX), content of the total copper (Cu) and zinc (Zn), measurement based on dry weight.

^d^: Measured value is below the limit of quantification (BLLOQ).

Table S2. Related primers of the standard PCR

| **Genes name** | | **Primers** | **References** |
| --- | --- | --- | --- |
| β-lactam ARGs | *bla_TEM_* | F: AGCATCTTACGGATGGCATGA | [1] |
|  |  | R: TCCTCCGATCGTTGTCAGAAGT |  |
|  | *bla_VIM_* | F: GCACTTCTCGCGGAGATTG | [1] |
|  |  | R: CGACGGTGATGCGTACGTT |  |
| MRGs | *copA* | F: TGCACCTGACVGGSCAYAT | [2-4] |
|  |  | R: GVACTTCRCGGAACATRCC |  |
|  | *czrC* | F: TAGCCACGATCATAGTCATG | [5] |
|  |  | R: ATCCTTGTTTTCCTTAGTGACTT |  |
| MGE | *IntⅠ1* | F: CTGGATTTCGATCACGGCACG | [2, 6, 7] |
|  |  | R: ACATGCGTGTAAATCATCGTCG |  |

References:

[1] Z.C. Zhou, W.Q. Feng, Y. Han, J. Zheng, T. Chen, Y.Y. Wei, M. Gillings, Y.G. Zhu, H. Chen, Prevalence and transmission of antibiotic resistance and microbiota between humans and water environments, Environ. Int. 121 (2018) 1155-1161.

[2] R. Zhang, J. Gu, X. Wang, Y. Li, J. Liu, C. Lu, L. Qiu, Response of antibiotic resistance genes abundance by graphene oxide during the anaerobic digestion of swine manure with copper pollution, Sci. Total Environ. 654 (2019) 292-299.

[3] H. Guo, J. Gu, X. Wang, X. Tuo, J. Yu, R. Zhang, Key role of cyromazine in the distribution of antibiotic resistance genes and bacterial community variation in aerobic composting, Bioresour. Technol. 274 (2019) 418-424.

[4] Y. Yin, J. Gu, X. Wang, W. Song, K. Zhang, W. Sun, X. Zhang, Y. Zhang, H. Li, Effects of Copper Addition on Copper Resistance, Antibiotic Resistance Genes, and intl1 during Swine Manure Composting, Front Microbiol 8 (2017) 344.

[5] E. Gomez-Sanz, K. Kadlec, A.T. Fessler, M. Zarazaga, C. Torres, S. Schwarz, Novel erm(T)-carrying multiresistance plasmids from porcine and human isolates of methicillin-resistant Staphylococcus aureus ST398 that also harbor cadmium and copper resistance determinants, Antimicrob. Agents Chemother. 57 (2013) 3275-3282.

[6] J. Zhang, T. Lu, Y. Chai, Q. Sui, P. Shen, Y. Wei, Which animal type contributes the most to the emission of antibiotic resistance genes in large-scale swine farms in China?, Sci. Total Environ. 658 (2019) 152-159.

[7] X. Qian, W. Sun, J. Gu, X.J. Wang, J.J. Sun, Y.N. Yin, M.L. Duan, Variable effects of oxytetracycline on antibiotic resistance gene abundance and the bacterial community during aerobic composting of cow manure, J. Hazard. Mater. 315 (2016) 61-69.

1. **Figures：**

| 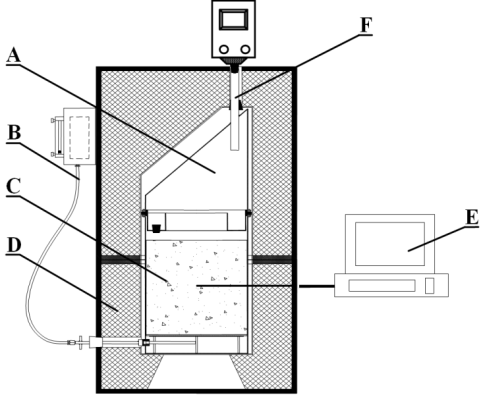 |
| --- |
| Figure S1. Schematic of the in-laboratory small-scale aerobic composting reactor system.  Note: (A) Upper part of the composting reactor; (B) ventilation control system; (C) composting materials; (D) insulation case; (E) temperature acquisition; (F) oxygen measurement device. |

|  |  |
| --- | --- |
| (a) Temperature | (b) TCB |
| Figure S2. Dynamic changes of (a) heaps and ambient temperature and (b) total culturable bacteria (TCB) during composting. | |

| 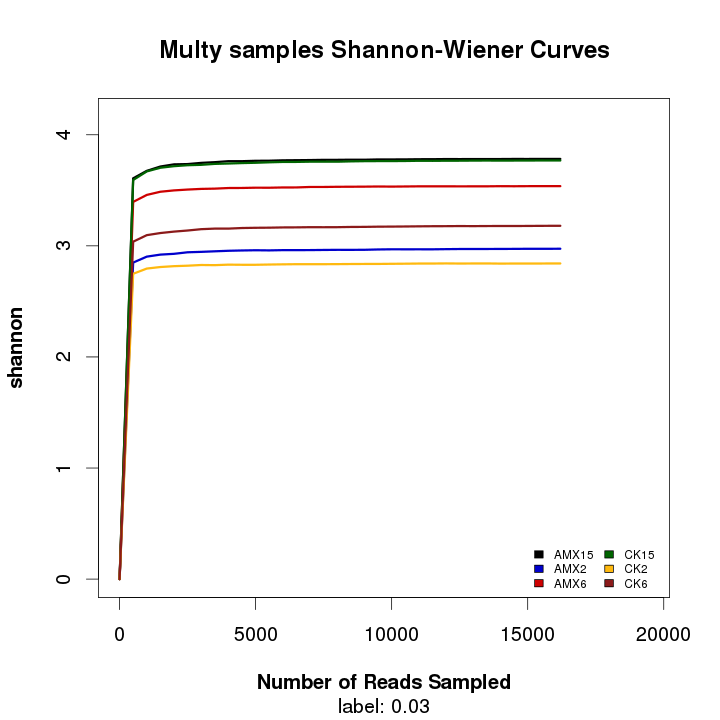 |
| --- |
| Figure S3. The Shannon-Wiener curves of the compost samples. |

| 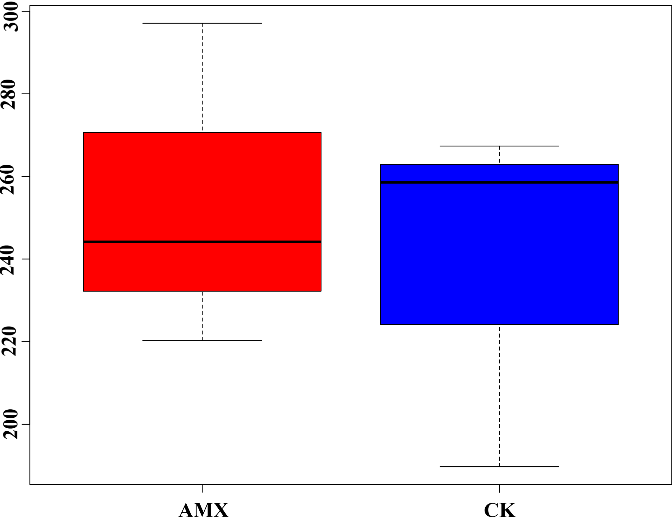  **(a)** | 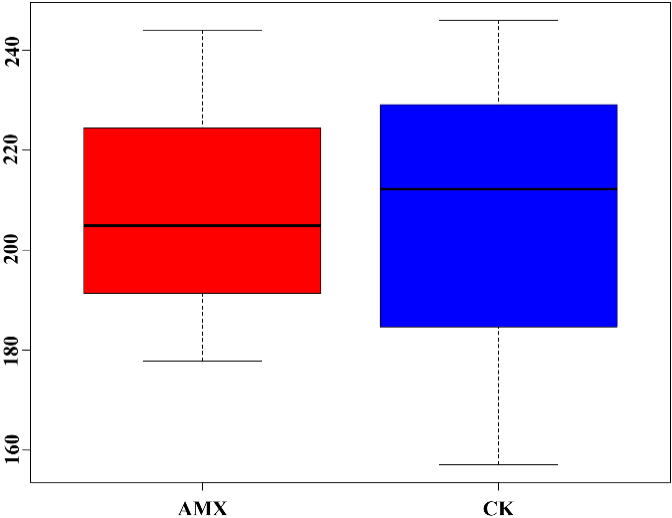  **(b)** |
| --- | --- |
| 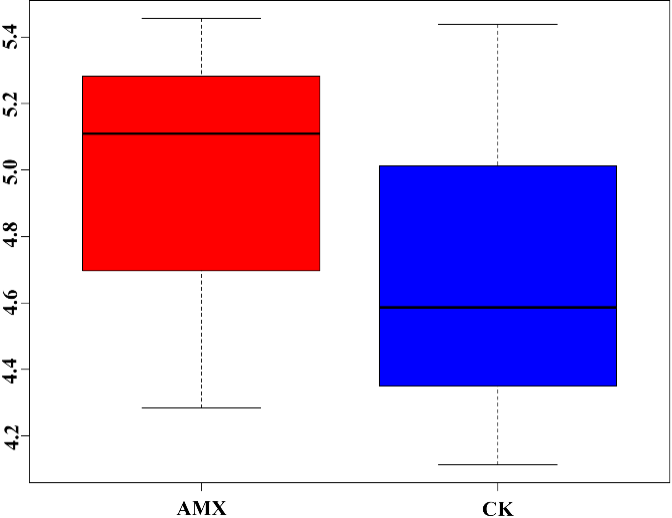  **(c)** | 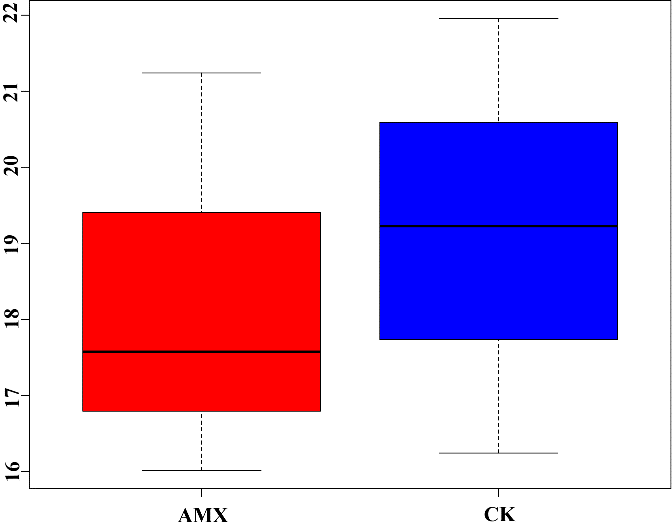  **(d)** |
| Figure S4. Boxplots of alpha diversity indexes of the compost samples: (a) Chao1, (b) Observed species, (c) Shannon, and (d) PD whole tree. | |


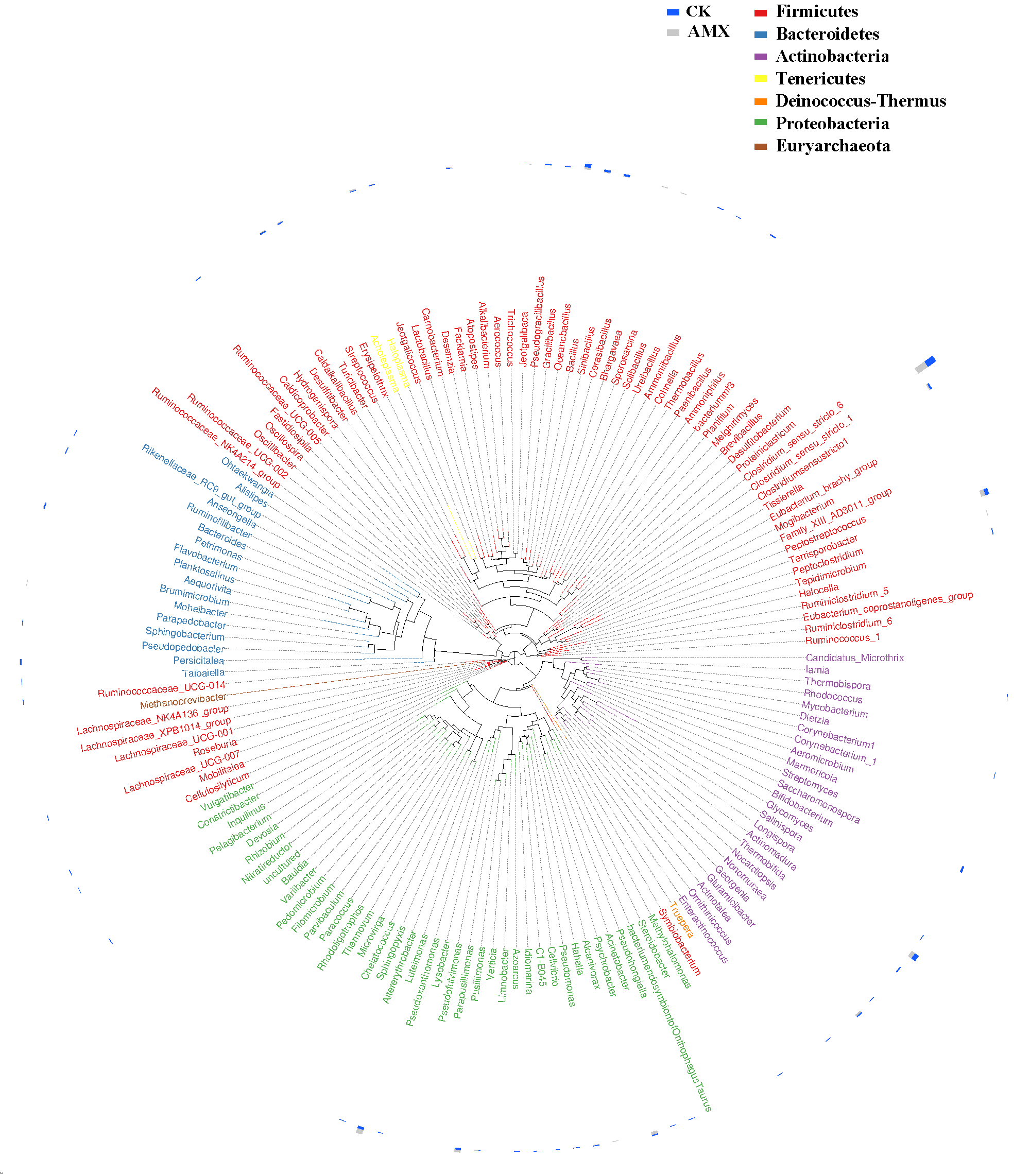


Figure S5. Genus-level phylogenetic tree of the compost piles.


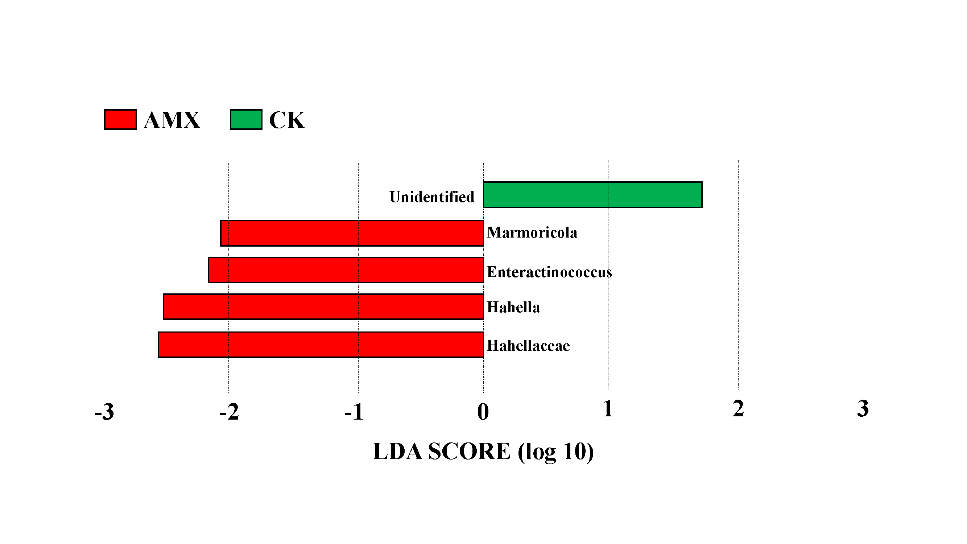


Figure S6. Difference analysis between two groups based on LDA distribution histogram.
